# Altered Resting-State Functional Connectivity in Wernicke's Encephalopathy With Vestibular Impairment

**DOI:** 10.3389/fneur.2019.01035

**Published:** 2019-09-27

**Authors:** Sun-Young Oh, Juhyung Lee, Jin-Ju Kang, Yeong-Hun Park, Ko Woon Kim, Jong-Min Lee, Ji-Soo Kim, Marianne Dieterich

**Affiliations:** ^1^Department of Neurology, School of Medicine, Chonbuk National University Hospital, Jeonju, South Korea; ^2^Research Institute of Clinical Medicine, Chonbuk National University Hospital, Chonbuk National University, Jeonju, South Korea; ^3^Department of Preventive Medicine, School of Medicine, Chonbuk National University Hospital, Jeonju, South Korea; ^4^Department of Biomedical Engineering, Hanyang University, Seoul, South Korea; ^5^Department of Neurology, Seoul National University Bundang Hospital, Seoul National University School of Medicine, Seoul, South Korea; ^6^Department of Neurology, Ludwig-Maximilians-University, Munich, Germany; ^7^German Center for Vertigo and Balance Disorders (IFB^LMU^), Ludwig-Maximilians-University, Munich, Germany; ^8^Munich Cluster for Systems Neurology (SyNergy), Munich, Germany

**Keywords:** Wernicke's encephalopathy, vestibule-ocular reflex (VOR), head-impulse test, functional connectivity, fMRI, vestibular cortex, insula

## Abstract

**Objectives:** To reveal the neural basis of Wernicke's encephalopathy (WE) with impaired vestibulo-ocular reflex (VOR), we evaluated resting-state functional connectivity (rs-fc) in the vestibular processing brain regions.

**Methods:** Rs-fc between the vestibular regions and the rest of the brain were compared with neurotological features including the head-impulse tests (vHIT) and caloric responses in patients with WE (*n* = 5, mean age 53.4 ± 10 years) and healthy controls (*n* = 20, mean age 55.0 ± 9.2 years). Rs-fc analyses employed a region of interest (ROI)-based approach using regions selected a priori that participate in vestibular processing including the cerebellar vermis, insula, parietal operculum, and calcarine cortex.

**Results:** The main neurologic findings for patients with WE were mental changes; gait ataxia; spontaneous and gaze-evoked nystagmus (GEN); and bilaterally positive HIT for the horizontal canals. Video HIT documented bilateral horizontal canal dysfunction with decreased gain and corrective saccades. Caloric irrigation and rotation chair testing revealed prominent bilateral horizontal canal paresis. Patients with WE also had decreased spatial memory, which substantially recovered after treatments. Functional connections at the predefined seed regions, including the insular cortex and parietal operculum, were attenuated in the WE group compared to healthy controls.

**Conclusions:** WE is related to impaired VOR and visuospatial dysfunction, and fMRI documented changes in the rs-fc of multisensory vestibular processing regions including the insula, parietal operculum, and superior temporal gyrus, which participate in integration of vestibular perception.

## Introduction

Wernicke's encephalopathy (WE) is an acute neuropsychiatric syndrome resulting from thiamine (vitamin B1) deficiency associated with many clinical conditions that impair thiamine absorption, including chronic alcohol abuse, gastrointestinal surgery, prolonged vomiting, chemotherapy, and systemic infectious and non-infectious diseases (1). The typical WE symptom triad consists of confusion, ocular abnormality, and gait ataxia. Findings of ocular abnormality include nystagmus, unilateral or bilateral palsy of any extraocular muscles and conjugate-gaze palsies, however, optic disc edema and retinal hemorrhages have also been reported in patients with WE (2). Additionally, vestibular dysfunction with an impaired horizontal vestibulo-ocular reflex (VOR) is another important feature of WE (1). Vestibular paresis in WE can be explained by lesions involving the vestibular nuclei (VN), especially the medial VN (MVN), nucleus prepositus hypoglossi (NPH), nodulus, and uvula (3, 4).

Slow (<0.1 Hz) spontaneous fluctuations in the blood oxygen level-dependent (BOLD) signal resemble a phase correlation within widely distributed functional networks, even in the absence of externally imposed tasks, i.e., during the “resting-state.” (5) Thus, resting-state functional connectivity magnetic resonance imaging (rs-fc MRI) is a non-invasive and robust measure of brain connectivity based on the observation that fluctuations in spontaneous, low frequency (<0.1 Hz) BOLD signals in spatially distant but functionally related brain regions are temporally correlated at rest (6). Rs-fc MRI can visualize and measure the brain's intrinsic functional architecture (7). Rs-fc among different regions and between established networks can be atypical in patients with certain brain diseases (8). However, few studies have investigated actual temporal changes in the functional connectivity of specific brain regions in patients with WE and impaired vestibular function. We hypothesized that atypical rs-fc in vestibular processing brain regions may be associated with vestibular dysfunction in WE. The aim of this study was to evaluate the functional connectivity of vestibular processing areas related to WE in the presence of vestibular paresis by using a well-established, data-driven neuroimaging analysis method.

## Methods

### Cases

#### Patient 1

**Figure 1:**
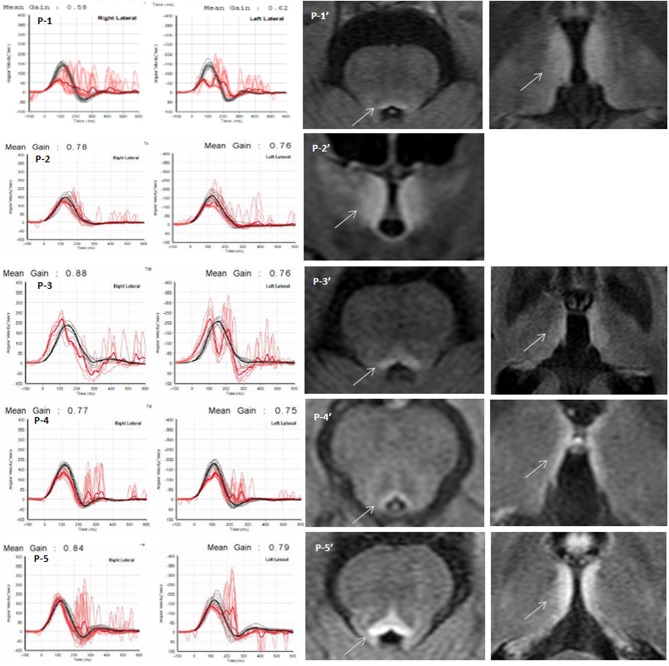
Quantitative video head impulse test (vHIT) and brain MRI. *Left panels*: Within 5 days after symptom onset, eye (in red) and head (in black) velocity traces for right and left head impulse tests of each Wernicke's encephalopathy patient are plotted against time (20 trials per canal) (P1~P5). Vestibulo-ocular reflex (VOR) gain (mean ± standard error) of each patient was significantly reduced, and overt catch-up saccades developed during passive head impulses. Note that eye velocity traces are inverted to allow for better visualization and comparison with head velocity traces. Mean gain values (eye velocity/head velocity) are shown in the upper part of the figure. *Right panels:* Brain magnetic resonance image (P1′~P5′) studies (fluid-attenuated inversion recovery images) show bilateral symmetrical lesions (white arrows) in the medial vestibular nucleus, periaqueductal region, around the hypothalamus, and periventricular regions of the thalamus.

A 65-year-old man was admitted due to dizziness, diplopia, and gait imbalance for 3 days. He had been a heavy drinker for more than 10 years with daily consumption of about 400 ml of the Korean liquor, Soju. Soju is the most popular liquor in Korea which is a clear, colorless distilled beverage made from rice with 17% ~ 53% alcohol content (alcohol by volume, ABV). Neurologic examination revealed an alert mentality but frequent inattentiveness and disorientation, dysarthria, and severe truncal ataxia with dysmetria in his upper arms. Spontaneous downbeat nystagmus was observed during with and without fixation, and horizontal and vertical gaze-evoked nystagmus were also detected. On saccadic commands, extraocular movement in all directions was markedly limited which did not improve with oculocephalic maneuvers. The video head-impulse test (vHIT) revealed decreased gain with bilateral catch-up saccades for both horizontal semi-circular canals (Figure 1, P-1). A bithermal caloric test revealed a severely decreased caloric response bilaterally (<5 deg/sec), and the patient had almost no response in the rotation chair test (time constant <3 s) (Table 1). Visuospatial memory was assessed during the acute period within a week after symptom onset but clear mentality, with block number, block design, and Corsi block tests, with results shown in Table 1. Blood thiamine level was 51 μg/ml (normal range 59–213 μg/ml) at admission. T2-weighted and FLAIR MRIs showed hyperintense lesions at the periaqueductal gray matter, medial thalami, and dorsal medulla (Figure 1, P-1′). Follow-up ocular examination and vHIT a week after thiamine supplementation (100 mg intravenously daily) showed normalized and visuospatial memory improvement (Table 1).

**Table 1:** Clinical findings of five cases with Wernicke's encephalopathy.

	**Patient 1**	**Patient 2**	**Patient 3**	**Patient 4**	**Patient 5**
**Age/sex**	**65/M**	**38/F**	**44/M**	**58/M**	**62/M**
Symptoms	Dizziness, diplopia, dysarthria, truncal ataxia, and dysmetria	Vertigo, severe ataxia, oscillopsia, apathy, and psychomotor slowing	Mental change and ataxia	Vertigo, ataxia, behavior change	Severe ataxia, bilateral dysmetria
Neurotologic findings	Spontaneous downbeat nystagmus, horizontal and vertical GEN, ophthalmoplegia	Spontaneous upbeat nystagmus, horizontal GEN, horizontal saccade limitation	Horizontal GEN, slow and limited horizontal saccades	Horizontal GEN	Horizontal GEN
Brain MRI (T2 or FLAIR, see Figure 1)	Increased signal in the mamillary bodies, medial thalamus, and periaqueductal area	Mild increased signal intensities in the medial thalami and periaqueductal gray matter	Mild-high signal intensities in the mammillary bodies, medial thalami	Increased signal in the dorsal medulla and pons	Increased signal in the medial thalami and periaqueductal gray matter
vHIT on HSCs (see Figure 1)	Decreased VOR gain (0.58/0.62) Catch-up saccades	Perverted (upward deviation of eyes and then downward movement) Catch-up saccades	Decreased VOR gain (0.88/0.76) Catch-up saccades	Decreased VOR gain (0.77/0.75) Catch-up saccades	Mildly decreased gain (0.84/0.79) Catch-up saccades
Caloric test	Bilaterally decreased response (<5 deg/sec)	No responses	Bilaterally decreased response	Bilaterally decreased response	Bilaterally decreased response
Rotation chair test					
VOR gain	Reduced gain	Reduced gain	Reduced gain	Reduced gain	n/a
Time constant	<3 s	2.5 s	4.5 s	(mean 0.195)	
Spatial cognition tests^*^					
Block number	6→8	7→8	7→7	6→8	5→7
Block design	5→6	5→7	6→9	7→7	6→8
Corsi block test	2→4	3→4	1→2	3→4	4→6

GEN, gaze-evoked nystagmus; vHIT, video head impulse test; HSCs, horizontal semicircular canals; VOR, vestibulo-ocular reflex.

* *Spatial cognitive tests at the acute period within 5 days of admission and 2 weeks after discharge*.

#### Patient 2

**Figure 2:**
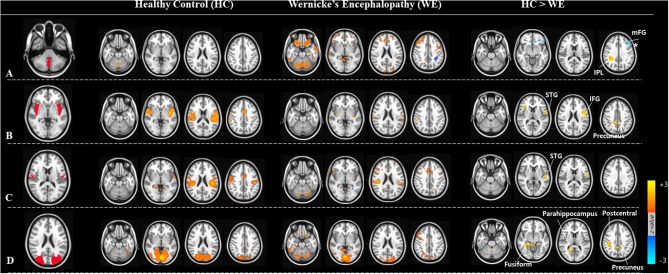
ROIs (region of interests) and the resting-state functional connectivity analysis. Four seed regions of **(A)** the posterior cerebellar vermis, **(B)** insular cortex, **(C)** parietal operculum, **(D)** visual cortex in the left panel. Group mean resting-state connectivity with four ROI seeds in healthy control (HC) and Wernicke's encephalopathy (WE) groups are shown in the middle panel. Subtraction of WE from HC revealed differences in functional connectivity between ROIs and other brain regions shown in the right panel.

A 38-year-old woman presented with a 1-month history of progressive vertigo, severe ataxia, oscillopsia, apathy, and psychomotor slowing. She was abstaining from food for religious reasons. Examination showed alert mentality but slight disorientation, perceptual disturbance, and impaired memory. Spontaneous upbeating nystagmus and horizontal gaze-evoked nystagmus were observed. Horizontal saccades were slow and limited (~30°) in both directions. The saccadic limitation did not improve with oculocephalic maneuvers. Horizontal vHIT revealed bilateral catch-up saccades (Figure 1, P-2), and perverted catch-up saccades, i.e., upward deviation of both eyes followed by downward movement. She showed bilateral limb dysmetria and severe truncal ataxia, and she could not stand unaided. Bithermal caloric irrigation revealed no response, and the sinusoidal rotation chair test showed significantly reduced gain and an increased VOR lead phase. Time constants for pre- and post-rotatory nystagmus decreased markedly (2.5 s, normal 14.6 ± 3.7 s) (Table 1). Visuospatial memory decreased during the acute phase of the disease and improved after treatment (Table 1). Brain MRI revealed signal intensities in the medial thalami and periaqueductal gray matter (Figure 1, P-2′). A week after thiamine treatment, follow-up vHIT normalized, and mental status, ocular signs, and ataxia recovered slowly.

#### Patient 3

A previously healthy 44-year-old man was admitted for mental change and ataxia. He had been a heavy drinker for several years, and recently, he had been fasting and drinking Soju for more than 10 days. Examination showed drowsy mentality with impaired orientation, increased deep tendon reflexes in both knees, bilateral dysmetria, and truncal ataxia. Horizontal saccades were slow and limited in both directions, and horizontal gaze-evoked nystagmus was observed. Horizontal vHIT revealed decreased gain and corrective saccades (Figure 1, P-3). Caloric response was reduced bilaterally, and pre- and post-rotatory nystagmus on rotation chair tests had short time constants (Table 1). On brain MRI, a high signal intensity in the periaqueductal gray and medial thalami was observed (Figure 1, P-3′). Three days after thiamine treatment, follow-up vHIT had normalized, and mental status, ocular signs, and ataxia recovered slowly. Visuospatial memory perception also improved after treatment (Table 1).

#### Patient 4

A 58-year-old man was admitted for vertigo, ataxia, and behavior change. He had been a heavy drinker consuming 600–1,000 ml of Soju daily. Neurological examination revealed impaired attention and truncal ataxia. He had no spontaneous nystagmus, but horizontal gaze-evoked nystagmus was observed. vHIT (Figure 1, P-4), bithermal caloric test, and sinusoidal rotation chair test showed significantly reduced horizontal VOR gain. Brain MRI showed high signal intensity in the dorsal medulla and pons (Figure 1, P-4′). A week after thiamine treatment, follow-up vHIT had normalized, and mental status, ocular signs, and ataxia recovered.

#### Patient 5

A 62-year-old man visited the hospital for severe ataxia and dizziness after consuming 1,000 ml of Soju daily for several months with no meal. Neurologic examination revealed gaze-evoked nystagmus, bilateral catch-up saccades on vHIT, dysmetria in both limbs, and severe truncal ataxia. On brain MRI, high signal intensity in the medial thalami and periaqueductal gray matter was observed (Figure 1, P-5′).

### Healthy Controls

We also examined 20 healthy, right-handed volunteers (11 males; mean age, 55.0 ± 9.2 years; range, 42–68 years old) with no history of neurotological or CNS disorders as a reference group for comparison with WE patients in secondary analyses. The patient and healthy control groups did not differ significantly in age (two-sample *t*-test, *p* = 0.81; degrees of freedom: 45) or sex (chi-square test *p* = 0.71).

The study was approved by the Institutional Review Board at Chonbuk National University Hospital, and all subjects gave their informed written consent.

No patient had their treatment (thiamine) delayed as a result of the investigation. Every patient had received iv thiamine treatment prior to undergoing their MRI scans and the scans were collected within 48 h after the initial treatment.

### Video-Head-Impulse Testing (vHIT)

vHIT (SLMED, Seoul, Korea) was conducted after calibrating eye position, and the technician applied a series of horizontal head impulses to the right and left in a random order. Vertical impulses were also applied along the right and left vertical canal planes (9). For each canal, 15 valid impulses were required and the VOR gain was calculated as the ratio of cumulative slow-phase eye velocity over cumulative head velocity from the onset of the impulse to the moment when head velocity returned to 0. We tried to maintain head displacements of 10–20° and head velocities at 150 and 200°/s. Corrective catch-up saccades were defined as saccades in the opposite direction of the head rotation that reached peak acceleration before (covert) or after (overt) the head stopped moving. We used cutoff values for VOR gains as based by the video goggle manufacturer data, i.e., 0.8 for the horizontal and 0.7 for the vertical canals. These values also agree with reported normative values for a wide range of ages (10).

### Visual Object and Space Perception Memory Test (Parts of WAIS-IV Test)

#### Block Number

Single or multiple dot stimuli were presented, and subjects were required to point to a number in an array that corresponded to the position of the dot. Ten test stimuli consist of two squares (62· × 62 mm), one above the other with a small gap. The top square contained randomly placed numbers from one to nine, and the bottom square had a single black dot corresponding to the position of one number. The task was to identify the number that corresponded with the position of the dot.

#### Block Design Test (BDT) (11)

This test required the patient to arrange a set of four or nine, two-colored blocks to duplicate a maximum of 10 target patterns presented in order of ascending difficulty.

#### Corsi Block Test

The examiner tapped cubes starting with a sequence of two blocks in front of the participant. Two trials were given per block sequence length. The subject had to tap the cube sequence in the same order immediately after the examiner finished.

### Imaging Data Acquisition and Management

Structural and functional images were acquired on a 3T MRI system (Magnetom Verio, Siemens Healthcare, Erlangen, Germany) with a 12-channel head coil. In a single session, 392 volumes (35 contiguous, axial, 3-mm-thick slices each; 1-mm gap) were acquired with a gradient echo, echo-planar imaging (EPI) T2^*^-sensitive sequence (repetition time, 2,000 ms; echo time, 30 ms; flip angle, 90°; matrix, 64 × 64; field of view, 192 × 192 mm). To reduce head movement and consequently artificial activation patterns, a foam pad was wrapped around the head. Structural images included a T1-weighted magnetization-prepared rapid gradient echo (MP-RAGE) sequence with a 256-mm field-of-view and 1.0 × 1.0 × 1.0 mm3 isotropic spatial resolution (TE, 4.37 ms; TR, 2,100 ms; 160 slices). Participants were instructed to minimize movement and keep their eyes closed but not fall asleep.

Resting-state fMRI data was preprocessed with AFNI software (http://afni.nimh.nih.gov/) (12). After discarding the initial five volumes from each fMRI, images were de-spiked, slice timing was applied, and head motion was corrected. In the head motion correction, all functional scans were realigned to the first image with a six-parameter, rigid body, spatial transformation, and differentiated head realignment parameters across frames yielded a six-dimensional time course that represents instantaneous head motion. The anatomical image was coregistered to the functional image by u sing affine registration with a Local Pearson Correlation cost function. All masks were also transformed to EPI space (13). The data were corrected with nuisance signal removed regression model with an anatomy based correlation correction (ANATICOR) method (14). The regressed images were temporally band-pass filtered (0.009 < f < 0.08) to reduce physiological noise. The filtered images were spatially smoothed by convolving the voxel values of the images with an isotropic Gaussian kernel of 6 mm width at half-maximum. Finally, the smoothed images were normalized to the Montreal Neurologic Institute space and resampled with an isotropic voxel size of 2 mm.

### Defining Regions of Interest (ROIs) and Seed-Based Functional Connectivity Analysis

The predefined established seed regions of the vestibular cortex in each hemisphere and the cerebellum (Figure 2, Table 2) were used to generate correlation maps in acute WE (*p* < 0.05, FWE correction). The regions of interest were chosen to represent the cortical network for vestibular processing and included the superior temporal cortex, insula, parietal operculum, primary visual cortex, and subcortically, the mammillary bodies and cerebellum. We delineated the structural boundaries of the whole seeds instead of defining spherical seeds within each region (Figure 2, the left column). The location of each ROI was modified manually until it was within the boundary of each seed region.

**Table 2:** Significant clusters in the two-sample *t*-tests comparing default mode functional connectivity in Wernicke's encephalopathy patients vs. controls.

**Group comparison**	**ROI (seeds)**	**Brain regions**	**Side**	**Coordinates (mm)**			**MW test *Z*-value**	**Voxels**
				**x**	**y**	**z**		
HC vs. WE	Posterior cerebellar vermis	Inferior parietal lobule	R	30	−40	30	2.535	752
		Middle frontal gyrus	L	−38	28	36	−2.366	331
	Insula	Inferior frontal gyrus	L	−54	8	26	2.535	625
		Superior temporal gyrus	R	44	10	−8	2.535	595
		Precuneus	L	−14	−52	38	2.535	378
	Parietal operculum	Superior temporal gyrus	L	−52	−18	4	2.535	339
	Calcarine cortex	Fusiform gyrus	R	38	−46	−12	2.535	655
		Postcentral gyrus	R	44	−28	38	2.535	425
		Precuneus	L	−8	−50	36	2.535	365
		Parahippocampal gyrus	R	24	−46	4	2.535	318

*Height and extent thresholds of p < 0.01 were used to determine significant clusters*.

Rs-fc analyses employed a ROI-based approach using a priori selected seeds that participate in vestibular processing. For each seed, a resting-state time series was extracted separately for each subject by computing the mean BOLD intensity of all voxels within the seed boundary at each MR frame (timepoint). A correlation map of each seed was obtained via correlation analysis between the seed reference time series and the time series of the rest of the brain in a voxel-wise manner. Then, a functional connectivity map was generated by converting correlation coefficients to *z*-values that represent the strength of functional connectivity between seeds by using a non-parametric two sample test (Mann Whitney *U*-test) [*P*-alpha <0.05 (uncorrected *p*-value <0.02 with 307 voxels)]. Thus, z-scores reflect to functional connectivity, and z-score maps were used in the correlation analysis. Connectivity maps were determined by correlating the mean time course of all voxel time courses within a brain mask.

## Results

### Neurotological Findings in Wernicke's Encephalopathy

Table 1 summarizes the behavioral and neurotological findings of our patients. The main neurologic findings were confusions with truncal ataxia and gait unsteadiness, spontaneous and gaze-evoked nystagmus, and positive clinical HIT for the horizontal canals. Caloric irrigation revealed bilateral horizontal canal paresis in all patients. vHIT revealed dominant or selective bilateral horizontal canal dysfunction with no or minimal vertical canal involvements (Figure 1).

The mean visuospatial memory assessment scores in healthy subjects were 8.15 ± 2.07, 8.38 ± 3.35, and 5.31 ± 1.25 for the block number, block design, and Corsi block tests, respectively (mean ± standard deviation). During the acute phase, WE patients had decreased spatial memory capacity in all three tests compared to healthy subjects (*p* < 0.01, Mann-Whitney *U*-test). The scores recovered substantially after treatment (Table 1).

### Resting-State Functional Connectivity in Wernicke's Encephalopathy

We obtained functional connectivity maps from each ROI in the healthy and patient groups, and they had different co-activation patterns. In WE patients, the connectivity strength of each vestibular processing ROI was lower than in healthy controls (Figure 2, middle columns). Subtraction analyses revealed that several FC between ROIs and other brain regions differed between the groups (Figure 2, right column and Table 2). The WE group had a weaker FC (Pα < 0.05 level) between the cerebellar vermis and inferior parietal lobule (IPL); between the insula and inferior frontal gyrus, superior temporal gyrus (STG), and precuneus; between the parietal operculum and STG; and between the calcarine visual cortex and other secondary visual cortex of fusiform gyrus, precuneus, and postcentral and parahippocampal gyrus. The WE group had a stronger rs-fc between the cerebellar vermis and middle frontal gyrus (Figure 2A, asterisk). Neither the WE nor the healthy control group had connectivity changes in the mammillary body. Connectivity strength was not related with age or education level in either group or with the duration or amount of alcohol consumption in the WE group (data not shown).

## Discussion

To better understand intrinsic brain connections in WE, we used a neuroimaging technique that measures resting-state functional connectivity (rs-fc) with functional MRI (fMRI). Compared to controls, WE with impaired vestibular and visuospatial cognition resulted in decreased connectivity between four different seeds—cerebellar vermis, insula, parietal operculum, calcarine cortex, and the rest of the brain. These results implicate the vestibular processing regions as a dysfunctional node causing functional deficits in a thiamine-deficient brain.

### Vestibular and Spatial Cognition Impairment in Wernicke's Encephalopathy

Typical lesions in WE are symmetrical in the periventricular areas in the thalamus, hypothalamus, mammillary bodies, periaqueductal region, floor of the fourth ventricle, and midline cerebellum (15). In addition to these areas, direct damage to the medial vestibular nuclei (MVN) and nearby nucleus prepositus hypoglossi (NPH) in the medulla is commonly associated with vestibulopathy and gaze-holding failure (16). Each of our patients had bilateral horizontal VOR failure on vHIT with gaze-holding nystagmus (GEN). Previous studies have proposed that the horizontal semicircular canals (HSCs) are predominantly involved in WE patients (17). MVN neurons are responsible for high-acceleration horizontal angular VOR (aVOR) and may be the most vulnerable to thiamine deficiency which cause a predominant horizontal VOR deficit. This selective susceptibility may result from the high metabolic demands of the neurons responsible for high-acceleration aVOR (1). Signals in the typical periventricular and periaqueductal locations of the dorsomedial brainstem and diencephalon superimposing the vestibular nuclei changed which explain the severe decrease of the horizontal VOR gain in these patients (Figure 1 and Table 1). In addition, our patients with WE had decreased short-term spatial memory, reflected by impaired visuospatial memory assessments during the acute phase (Table 1).

Based on these findings, patients with thiamine deficiency may present with acute or subacute vestibular symptoms especially with loss of bilateral horizontal VOR function and impaired spatial cognition as a sign of encephalopathy. A growing body of evidence is providing insight into vestibular contributions to a variety of cognitive processes, including visuospatial and perceptual ability, attention, memory, and executive function. With concomitant impaired performance on several aspects of the virtual Morris Water Maze test without impairment in intelligence or non-spatial memory, significant atrophy in hippocampal volume (16.9%) has been reported in patients with chronic bilateral vestibular impairment (18). Another studies with surgical vestibular deafferentation showed increased numbers of turn errors and time required to reach memorized targets when walking, particularly during eyes closed navigation, i.e., without visual cues (19). These results suggest that in regard to spatial cognition, patients with vestibular deficits could have impairment in their spatial navigation abilities including the WE patients with thiamine-deficient induced vestibular deafferentation.

### Resting-State Functional Connectivity Changes in Wernicke's Encephalopathy

Although typical WE lesions involve the brainstem and periventricular area, WE patients frequently have symptoms that extend beyond the brainstem, such as memory and visuospatial dysfunction (20). A previous fMRI study revealed that the fc strength of the mammillothalamic pathway differed significantly between chronic WE and healthy controls. This fc strength significantly correlated with memory function (21). WE have defective blood-brain barrier mainly in the periventricular regions; however, signal intensity can change in atypical regions including the cerebral cortex, cranial nerve nuclei, red nuclei, dentate nuclei, caudate nuclei, splenium, and cerebellar vermis. These atypical changes may lead to diverse subcortical and cortical cognitive dysfunctions (22). Recently functional neuroimaging studies in humans have used to identify widely distributed vestibular cortices. Vestibular responses in the posterior insula corresponding to the parieto-insular vestibular cortex (PIVC) of the monkey, showed increased neural activity (fMRI) during various vestibular (e.g., caloric, galvanic) stimuli in healthy human subjects (23). The middle and posterior insula, inferior and posterior parietal cortex, posterior STG, somatosensory cortex, cingulate gyrus, frontal cortex, and hippocampus also belong to multisensory vestibular processing cortical areas (24–27).

In the current study, WE patients had impaired VOR and vestibular deafferentation with the MVN and NPH lesions in the brainstem which caused vestibular cortical hypofunction reflected by decreased rs-fc in the vestibular processing regions. Rs-fc decreased in regions that are core for vestibular cortical processing and integrating multisensory signals into a perception of spatial orientation and self-motion (28–30). Therefore, dysfunction in these areas might be linked with spatial and other higher vestibular cognitive dysfunctions. These changes were found without any vestibular stimulation, which indicates a fundamental, disease-related change in the resting brain, i.e., a change in resting-state connectivity. A recent rs-fMRI study of patients with unilateral vestibular neuritis showed decreased functional connectivity in the intraparietal sulcus and supramarginal gyrus in IPL (31). And, PET (32) and rs-fMRI (33) studies of bilateral vestibulopathy patients revealed that reduced task-independent metabolism in the posterior insula and lower bilateral functional connectivity in the posterior insula and parietal operculum. Evidence shows that thiamine-deficient animals perform worse in spatial cognition tests such as the Morris Water Maze (34, 35). In a previous fMRI study using parametric sound stimuli, vestibular, and auditory sense convergence was observed in overlapping regions of the caudal part of the superior temporal gyrus (STG) and posterior insula (27). In addition, some regions only responded to stimulation above the vestibular threshold, suggesting that vestibular (otolith) signals are processed in the inferior insula, IPL, and cerebellum (27). These data indicate that the STG, insula, and IPL, including the parietal operculum, could contain information for multisensory vestibular integration, i.e., an important hub for higher vestibular cognition tasks such as spatial localization (27, 30). In another words, these brain areas are part of a complex neural network for vestibular and visuospatial processing and memory (36, 37).

In addition to decreased rs-fc among the vestibular regions, patients with WE had increased connectivity between the middle frontal gyrus and cerebellum (Figure 2A, asterisk). Patients with WE usually have microscopic cerebellar degeneration (38) that could contribute cerebellar dysfunction. The cerebellum (Crus I) is functionally connected to the dorsolateral prefrontal and middle frontal cortices (BA 9, 46), pre-supplementary motor area, and anterior cingulate cortex (39). Furthermore, transcranial direct-current stimulation (tDCS) suppresses cerebellar excitability and disinhibits frontal activity (40). Therefore, WE patients with VOR dysfunction who have cerebellar pathology might show the increased fc between the cerebellum and frontal cortex. While sample sizes of WE patients (*n* = 5) in the current study were small, findings provide insight into the vestibulo-cortical connections that are disrupted with vestibular deafferentation (horizontal VOR impairment due to brainstem lesions), and that manifest as impaired visuospatial cognition and mental representation in the three-dimensional space. Further study examining the structural and functional neuroimaging characteristics of patients with vestibular impairment would provide insight into the mechanism of vestibular-related visuospatial impairment.

## Data Availability Statement

The datasets generated for this study are available on request to the corresponding author.

## Ethics Statement

The studies involving human participants were reviewed and approved by Institutional Review Board at Chonbuk National University Hospital. The patients/participants provided their written informed consent to participate in this study. Written informed consent was obtained from the individual(s) for the publication of any potentially identifiable images or data included in this article.

## Author Contributions

S-YO, J-SK, and MD conceived and planned the research. Y-HP and J-SK contributed to analyze the image data. J-JK and JL took the lead in writing the manuscript. All authors provided critical feedback and helped shape the research, analysis, and manuscript.

### Conflict of Interest

The authors declare that the research was conducted in the absence of any commercial or financial relationships that could be construed as a potential conflict of interest.
